# Voltage-gated ion channels in cultured gill epithelia of rainbow trout, *Oncorhynchus mykiss*, change in transcript abundance with exposure to freshwater

**DOI:** 10.1016/j.cbpa.2025.111835

**Published:** 2025-03-10

**Authors:** Siaje Gideon, Brendan Boyd, Brandon Ramirez Sierra, Dennise Arenas, Perla Ochoa, John Eme, Dennis Kolosov

**Affiliations:** Department of Biological Sciences, California State University San Marcos, CA 92096, USA

**Keywords:** Fish osmoregulation, Gill epithelium, Voltage-gated ion channels

## Abstract

Salmonid fishes are well adapted to transition between salinities as part of a diadromid lifestyle, and many species are both economically and environmentally important. Ion-transporting gill epithelium helps fishes maintain ion balance during salinity transition. Recent transcriptomic surveys suggest that voltage-gated ion channels (VGICs) are present in gill epithelium of fishes. However, fish gill epithelia are architecturally complex and structurally heterogeneous (which includes layers of excitable tissues), which necessitates a model to study isolated gill epithelial cells. In the present study, we isolated gill epithelial cells, used them to reconstruct primary cultured gill epithelium model, and exposed the reconstructed epithelia to apical freshwater (FW). Using RNAseq and molecular biology we demonstrate that multiple VGICs are expressed in cultured gill epithelia of a salmonid, rainbow trout *Oncorhynchus mykiss*. Following apical exposure to FW, multiple subunits of voltage-gated calcium (Ca_V_) channels, as well as KCNE2 were upregulated in mRNA abundance. Using a custom-made antibody, we demonstrated that Ca_V_1.3 immunolocalized to the apical membrane of epithelia in intact trout gill, as well as in the cultured gill epithelium. Pharmacological inhibition of Ca_V_1 in FW-exposed cultured epithelia led to increased transepithelial resistance. Therefore, we propose that VGICs are present in gill epithelia of fishes, and may rapidly and autonomously respond to environmental salinity changes to help the fish maintain salt and water balance, where Ca_V_1 specifically may play a particularly important role in rapid adjustment of gill epithelia barrier properties and resistivity and potentially in responding to regulatory cell volume decrease in vitro.

## 1. Introduction

Animals rarely live in environments that perfectly match the salt and water content of their bodies. Because of this, they employ specialized barrier tissues called epithelia that line osmoregulatory organs (e.g., gut, kidney, skin, gill) to interact with the environment and transport ions and water in and out of the body. Animal epithelia (e.g., the fish gill) are multifunctional tissues responsible for salt and water balance, nitrogenous waste excretion, and acid-base balance (e.g., Evans et al., 2005). Epithelia generally work by actively transporting ions across the tissue (which usually draws water osmotically if aquaporins are present), while tightly regulating and fine-tuning paracellular permeability (Epstein et al., 1983). Although a lot is known about how paracellular and transcellular pathways contribute to ion transport in epithelia (e.g., Jonusaite and Himmerkus, 2024; Dow et al., 2021), very little is known how these processes are regulated at the molecular level in response to rapid changes in external salinity, and more specifically to the rapid mechanisms of ion transport regulation in epithelia.

Our recent research on animal epithelia has shown that epithelia of insects are capable of rapidly (~10 min) responding to salt and water imbalance using voltage-gated ion channels (VGICs) (Kolosov et al., 2018a; Kolosov et al., 2018b; Kolosov et al., 2019; Kolosov and O’Donnell, 2019; Kolosov and O’Donnell, 2022). VGICs have been detected in vertebrate epithelia (see below), but studies connecting them to osmoregulation are lacking, and none have been published for the fish gill epithelium specifically. VGICs open and close in response to changes in membrane potential and demonstrate very fast channel kinetics typically associated with fast-response excitable tissues (e.g., nerves and muscles). Several VGICs in non-innervated and non-contractile epithelia have already been shown to directly regulate ion transport - e.g., voltage-gated Ca^2+^ channel (Ca_V_1) and hyperpolarization-activated cyclic nucleotide gated (HCN) channels connect membrane potential and K^+^ transport via Ca^2+^ signaling in insect epithelia (Kolosov and O’Donnell, 2019; Kolosov et al., 2021; Farrell et al., 2024).

There is no central paradigm/model explaining how VGICs are used by animal epithelia. Indeed, recent studies demonstrate osmoregulatory epithelia of all animals examined to date express VGICs, supporting their important role in rapid detection of environmental or systemic changes in ion and water content (reviewed in Kapoor et al., 2021; Dates and Kolosov, 2024). VGICs could allow vertebrate epithelia to respond to acute osmoregulatory imbalance, providing an advantage for these “outward-facing” epithelia that would experience the imbalance first (before initiating a systemic response). Cutting-edge progress has been made recently dedicated to deciphering the mechanisms of epithelial osmosensing (e.g., Mergler et al., 2012; Kültz, 2015; Siroky et al., 2017), ion transport (e.g., Choudhury et al., 2022) and barrier regulation (Günzel and Yu, 2013). In contrast, despite the fact that voltage-gated H^+^, Na^+^, K^+^, Ca^2+^, transient receptor potential (TRP) and HCN channels have all been detected in animal epithelia (see Pitt et al., 2021; Abbott, 2014; Li et al., 2018; Barshack et al., 2008; Bleich and Warth, 2000; Demolombe et al., 2001; Morera et al., 2015; Yang and Cui, 2015; Zhu et al., 2010; Yao et al., 1996; Grunnet et al., 2003; Girault and Brochiero, 2014; MacPherson et al., 2001; Bolívar et al., 2008; Schlingmann et al., 2002), the functions of most in the regulation of epithelial ion transport and barrier properties remain unstudied to date.

The fish gill epithelium presents an attractive model for the study of VGICs in vertebrate epithelia. Fishes are the most diverse and successful clade of vertebrates, demonstrating extreme adaptation for salinity acclimation and tolerance, which requires rapid sensing of environmental salinity (Kültz, 2015). In freshwater (FW) fishes, the fish gill actively takes up ions from FW and restricts paracellular loss of ions back into freshwater. The gill is a structurally heterogeneous and architecturally complex organ that consists of several tissue layers, including muscles and nerves that also express VGICs (Evans et al., 2005). The gill epithelial cells can be separated from the gill and primary cultured gill epithelia grown in vitro to allow detection of VGICs. This model has been used for over 30 years, and in the current study can help quantify the autonomous response of only the gill epithelium to a change in environmental salinity, eliminating confounding variables of additional tissue layers and systemic hormonal support (Pärt et al., 1993; Gilmour et al., 1998; Wood and Pärt, 1997; Wood et al., 1998; Kelly et al., 2000; Chasiotis et al., 2010; Kelly et al., 2000; Sandbichler et al., 2011; Kolosov et al., 2014; Kolosov and Kelly, 2017). This model mimics passive and active transport and electrophysiology properties of the intact gill epithelium, but consists exclusively of the pavement cells (Kelly et al., 2000), as opposed to another model which requires a distinctly different methodology and can include mitochondrion-rich cells (Fletcher et al., 2000; Schnell et al., 2016). When exposed to FW apically (on the water-facing side), these cultured epithelia respond with altered TER, altered tight junction permeability, and a regulatory cell volume decrease using Ca^2+^ signaling (Wood et al., 1998; Leguen and Prunet, 2001; Kelly and Wood, 2002; Wood et al., 2002).

In the current study, we used RNAseq and molecular biology tools in primary cultured rainbow trout (*Oncorynchus mykiss*) gill epithelia to uncover which VGICs are present, and which respond to apical FW exposure with increased mRNA abundance. The objectives were to investigate whether: (i) VGICs are expressed in the cultured gill epithelia of rainbow trout, (ii) some VGICs alter in mRNA abundance when epithelia are exposed to FW, (iii) Ca_V_1 is expressed in the gill epithelia as this VGIC has been shown to regulate ion transport in epithelia of several animals to date (Kolosov et al., 2021; Farrell et al., 2024), and (iv) Ca_V_1 may be involved in the response to FW. Using RNAseq and molecular biology, we demonstrated that multiple VGICs are expressed in the primary cultured gill epithelia, and that abundance of several channels was altered when epithelia were exposed to FW. Using immunohistochemistry, we have showed that Ca_V_1 is found in primary cultured and intact whole-gill epithelia. By pharmacologically inhibiting Ca_V_1 following exposure to FW, we demonstrated that primary cultured gill epithelia exhibited alterations in transepithelial resistance (TER), suggesting Ca_V_1 may specifically contribute to the response of cultured gill epithelia to FW. This is the first study to demonstrate that VGICs in the gill epithelium of fishes can meaningfully respond to apical FW exposure and alter resistive properties of the cultured gill epithelia.

## 2. Materials and methods

### 2.1. Experimental animal husbandry

Several thousand freshly fertilized Shasta strain diploid rainbow trout embryos (*Oncorhynchus mykiss* (Walbaum 1792), were obtained from Mount Shasta Hatchery, CA and shipped to California State University San Marcos, CA overnight. Embryos were incubated at 7 °C, and several hundred subsequent hatchlings were grown to juvenile size (12–15 cm) in 25-L tanks at 13 °C and 12 h light: 12 h dark photoperiod in filtered dechlorinated ion-poor FW (composition in μM: 19 Na^+^, 44 Cl^−^, 2.3 K^+^, 584 Ca^2+^, pH = 7.5). The FW was prepared by mixing 1 part of dechlorinated tap water with 3 parts of distilled water (local San Diego tap water in is very hard). A subset of juveniles (*N* = 10) was transferred to 80-L opaque polyethylene tanks maintained at 13 °C (Aqua Ero USA Max-Chill). Water was tested weekly and changed weekly to ensure appropriate water quality parameters - pH as indicated above, while ammonia and nitrite levels were kept at 0 ppm (ppm), and nitrate levels were kept at 0–5 ppm. All experimental procedures and animal care were conducted in accordance with protocol #22–010 approved by the California State University San Marcos Institutional Animal Care and Use Committee protocol.

### 2.2. Preparation of primary cultured gill epithelia

Prior to dissection, fish were net-captured and placed into 1 L of tricaine methanesulfonate (TMS) anesthesia solution (0.25 g/L) for ~10 min. Once anesthetized, a spinal transection was performed to euthanize the fish. 30 mL of sterile phosphate-buffered saline (PBS, pH = 7.7) was used to perfuse the gill arches by inserting the syringe needle into the bulbus arteriosus of the fish heart after accessing it ventrally.

A primary cultured gill epithelium model was prepared using methodology developed by Wood and Pärt (1997) and described in detail by Kelly et al. (2000). This model used in this study has been characterized in multiple studies to be composed exclusively of gill pavement cells (Kelly et al., 2000; Kolosov et al., 2014). Briefly, after perfusing the gills with PBS (pH = 7.7), the operculum was removed to expose the gill arches, which were dissected out in room temperature sterile PBS (pH = 7.7). Excess mucus and blood clots were removed using Kim wipes, and gill arch filaments were separated from cartilage, cut into smaller pieces ~1/3 gill filament in size. Dissected gill arches were treated with antibiotic/antimycotic solution (10 min in Cytiva 100 U/mL Penicillin G, 100 μg/mL Streptomycin, 0.25 μg/mL Amphotericin B). Gill epithelial cells were isolated by three successive rounds of trypsination coupled with manual agitation. Gill epithelial cells were collected using a Corning 100-μm nylon cell strainer, counted using hemocytometer, and seeded into 25-cm^2^ cell culture flasks in Leibovitz’s L-15 culture medium (L15, ThermoFisher Scientific Cat#11415–064, Lot#2766630) supplemented with antibiotics/antimycotics and 6 % fetal bovine serum (FBS, ThermoFisher Scientific Cat#26140–079, Gibco, qualified, US origin, Lot#2490721RP) with the following ion composition (in mM): 140.61 Na^+^, 147.76 Cl^−^, 5.77 K^+^, 1.26 Ca^2+^. As indicated in the original methodology, FBS is used to provide to provide factors that aid in the development of the parameters of a mature cultured epithelium (examples of established fish gill epithelial models that use FBS include – rainbow trout est. by Pärt et al., 1993; goldfish est. by Chasiotis and Kelly, 2011; puffer fish est. by Bui et al., 2010). Media changes were performed at 1 day post-seeding in flask-cultured epithelia, and then every 48 h until the end of culture period.

Following a 6-day period of flask culture, epithelia were harvested by trypsination and seeded onto semi-permeable polyethylene terephthalate filter inserts (0.9 cm^2^ area, 0.4 μm pore; BD FalconTM, Fisher Scientific, Carlsbad, CA, USA). Companion cell culture plates (Falcon BD) were used to keep filter inserts immersed in L15 on both sides (L15/L15 symmetrical conditions) as epithelia were developing transepithelial resistance (TER). L15 was supplemented with antibiotics/antimycotics (Cytiva 100 U/mL Penicillin G, 100 μg/mL Streptomycin, 0.25 μg/mL Amphotericin B) and 6 % FBS.

### 2.3. Media changes, TER measurements, and apical FW exposure

Media changes were performed at 1 day post-seeding in insert-cultured epithelia, and then every 48 h until the end of culture period. Chopstick electrodes (STX-2) attached to an EVOM^2^ epithelial voltohmmeter (World Precision Instruments, Sarasota, FL, USA) were used to monitor TER in insert-cultured epithelia. TER was measured daily in insert-cultured epithelia, and measured TER values were background corrected by subtracting TER of a blank insert containing only media of appropriate composition. All TER measurements are expressed in Ω*cm^2^.

L15-cultured epithelia were allowed to develop stable TER (typically occurs after 5–7 days as confluence and resistive properties of the newly formed tight junctions in the epithelial layer mature). Once, stable TER is developed, the cultured epithelium is deemed mature, and experiments can be conducted on it for several days without conflating developmental increase in TER with that observed because of experimental treatment. Following this, apical medium was replaced with temperature-equilibrated filter-sterilized (0.2 μm pore size) FW obtained from the holding tank (see composition above) after several rinses with FW to ensure complete removal of residual L-15 media. FW-exposed epithelia were allowed to develop stable TER (measured upon introduction of FW, and every 30 min thereafter). After 2 h of FW exposure, L15-cultured and FW-exposed epithelia were harvested for total RNA isolation following a complete removal of media from the apical side.

### 2.4. RNA extraction and purification

RNA extraction was performed according to Kolosov et al., 2018a, 2018b. Briefly, after aspiration of apical media, insert-cultured epithelia were immersed into 0.5 mL of TRIzol reagent (ThermoFisher Scientific). Epithelia suspended in TRIzol were collected, flash-frozen on dry ice, and stored at −80 °C until processed.

RNA extraction (RNA precipitation, wash and redissolving) was performed using TRIzol reagent according to the manufacturer’s instructions as described in Kolosov et al. (2014). Briefly, epithelia were homogenized in TRIzol using a 26G syringe needle and allowed to remain at room temperature for 5 min to dissociate nucleoprotein complexes. Molecular grade chloroform (100 μL) was added to every sample. All samples were vigorously agitated, incubated at room temperature for 3 min, and centrifuged to perform phase separation, after which the clear aqueous phase containing total RNA was collected into a set of sterile 1.5 mL Eppendorf tubes on ice. Total RNA was precipitated with isopropanol, supernatant was removed from every tube by aspiration and RNA pellets were washed in 75 % molecular grade ethanol solution in RNase-free water, and centrifuged again to immobilize pellets. Ethanol was then removed by aspiration, and pellets were left to air-dry for 1 min at room temperature, after which they were dissolved in 12 μL of RNase-free water. Total RNA was purified using a Thermo Scientific GeneJet RNA Cleanup and Concentration Micro Kit according to manufacturer’s instructions (cat. no. K0841, ThermoFisher Scientific). The concentration and quality of purified RNA were determined using a nano-sample spectrophotometer (DeNovix DS-11, Wilmington, DE, USA). Absorbance ratios of A260/A280 = 2.034 ± 0.001 and A260/A230 = 2.061 ± 0.047 were observed, indicating excellent sample purity.

### 2.5. RNAseq library preparation, sequencing, quality control, mapping and differential expression analysis

Total purified RNA from 3 biological replicates for each treatment group (L15-cultured and FW-exposed) was sent to University of California Riverside Genomics Facility for Illumina library preparation. Single-end 100-bp Illumina libraries were prepared by the University of California Riverside Institute for Integrative Genome Biology sequencing facility (UCR IIGB, Riverside, CA, USA) using the following methodology. Total RNA samples were screened with an Agilent Bioanalyzer 2100 to confirm quality and concentration. Each sample was diluted to 250 ng for input into NEBNext Poly(A) mRNA Magnetic Isolation Module (E4790, New England Biolabs), followed by NEBNext Ultra II Directional RNA Library Prep kit for Illumina (E7760). The NEB protocol was followed with the following adjustments: 0.8× bead clean after second strand synthesis, adaptor dilution of 1:30, 0.7× bead clean after ligation, 15 cycles of amplification, and dual bead clean after enrichment. Standard Illumina adaptors were used for all, but each library was barcoded independently with TruSeq indexes. Final libraries were qualified and quantified with Agilent Bioanalyzer 2100, then pooled equimolarly. Sequencing was performed with Illumina Next-Seq500 using a High-output Single-end 100-bp kit.

Sequencing (at UCR IIGB) of six strand-specific libraries (3 L15-cultured and 3 FW-exposed) with 100-bp single-end reads yielded a total of 665,926,272 reads with an average of ~89.28 million raw reads per library. Quality control (QC) reports were performed by UCR IIGB, and reads were subjected to quality control trimming with Trimmomatic using previously described protocols (usegalaxy.org; Goecks et al., 2010; Kolosov et al., 2019). Raw sequencing data will be available at SRA BioProject upon acceptance of manuscript for publication.

The USDA OmykA1.1 genomic transcriptome was accessed at ncbi.nlm.nih.gov, and official genome transcript annotation was used for mapping. Mapping of Next Generation Sequencing (NGS) reads to this transcript set was performed using Salmon (Galaxy version 1.3.0, useg alaxy.org) mapping software (Patro et al., 2017). Mapping was performed to the *O. mykiss* transcriptome, and annotation supplied with the official genome annotation was used.

Differential expression analysis was performed in R (version 4.4.1) using the DESeq2 package (release version 1.44.0) (Love et al., 2014), where the FW-exposed group was compared with the L15-cultured group. This analysis involved compiling counts tables for all samples generated by Salmon in Galaxy, uploading them to R, creating a meta-data table indicating which sample belongs to which treatment group, and running the DESeq2 script in R as instructed in the vignette publication. DESeq2 package estimates size factors and dispersions, determines mean dispersion relationship, and builds a model of differential expression. DESeq2 analysis in R resulted in a list of all differentially expressed transcripts (Supplementary Table S1) accompanied by the log_2_ fold-change (log_2_FC), *P*-value of the change (P), and P-value adjusted (P_adj_) for the false discovery rate (FDR) by the DESeq2 Benja-mini–Hochberg algorithm. For further information, the reader is directed to Love et al. (2014).

### 2.6. Synthesis of cDNA, PCR, gel electrophoresis, and qPCR amplification of VGIC transcripts

The FW exposure, RNA extraction and purification protocol described above was used to harvest an additional set of L15-cultured (*n* = 7) and FW-exposed (*n* = 11) epithelia. 1 μg of total RNA from each sample was treated with DNAse and used for oligo-dT- aided reverse-transcriptase cDNA synthesis. Using transcript-specific primers indicated below, cDNA was diluted in RNAse/DNAse-free water and used for PCR detection of VGIC expression and qPCR quantification of transcript abundance.

Analysis of RNAseq data suggested that multiple VGICs were expressed in the primary cultured epithelia of rainbow trout. Expression of transcripts encoding VGICs was confirmed by RT-PCR in cultured trout gill epithelia using three replicates and DreamTaq Hot-Start Supermix (ThermoFisher Scientific, Carlsbad, CA, USA) in a BioRad PCR machine for RT-PCR (PTC-2000; Bio-Rad Laboratories Canada Ltd., Mississauga, ON, Canada). Changes in transcript abundance in cultured epithelia exposed to FW were assessed using quantitative real-time PCR (qPCR) with 2× DyNAmo Flash SYBR Green qPCR Mastermix (F415L, ThermoFisher Scientific) and ThermoFisher Scientific QuantStudio 3 qPCR machine running ABM Design and Analysis software. Primers specific to VGICs were designed based on sequences obtained from RNAseq data. Gel electrophoresis was performed on PCR amplicons to confirm their size, and electrophoresed samples were subsequently extracted from the gel, purified and sequenced to ensure amplicon identity (see Table 1 for PCR primer and cycling information).

The following reaction conditions were used: 1 cycle for denaturation (95 °C, 4 min), followed by 40 cycles of: denaturation (95 °C, 30 s), annealing (see Table 1, 30 s) and extension (72 °C, 30 s); and a final extension step (72 °C, 10 min). To ensure that a single PCR product was synthesized during reactions, a dissociation curve analysis was carried out after each qPCR run. Transcript abundance was normalized to that of *O. mykiss* elongation factor α1a (*ef*). The use of *ef* for gene of interest normalization in FW exposure studies was validated by statistically comparing *ef* threshold cycle values between epithelia to confirm that no statistically significant changes occurred (*p* = 0.3947, df = 16, Student’s 2-tailed *t*-test, GraphPad Prism version 10.4.0).

### 2.7. Immunohistochemistry and immunolocalization of Ca_V_1 in intact cultured epithelia and sectioned whole gill epithelia of rainbow trout

Apical media were removed from insert-cultured rainbow trout gill epithelium preparations, after which they were rinsed and subsequently fixed with 3 % paraformaldehyde (PFA) for 20 min at room temperature (pH = 7.4). The inserts were then rinsed with ice-cold PBS (pH = 7.4) twice and permeabilized in methanol at −20 °C for 5 min. The methanol was then aspirated, and the apical side was washed 3 times for 5 min with cold PBS (pH = 7.4). The PBS (pH = 7.4) was then aspirated and 500 μl of Triton X-100 (0.01 % in PBS, pH = 7.4) was added to the apical side and allowed to sit at room temperature for 10 min. The Triton-X 100 was then aspirated out and then the apical side was washed twice for 5 min at room temperature with PBS (pH = 7.4). The PBS (pH = 7.4) was aspirated and then the inserts were incubated with antibody dilution buffer (ADB; 10 % goat serum, 3 % bovine serum albumin and 0.05 % Triton X-100 in PBS, pH = 7.4) for 1 h at room temperature. The ADB was then aspirated, and the inserts were probed overnight with a custom-synthesized rabbit anti-Ca_V_1.3 polyclonal antibody (epitope – CWSEYDPDAKGRIKH; GenScript, Piscataway, NJ, USA; validated in Farrell et al., 2024) diluted 1:800 in ADB overnight in a dark drawer at room temperature.

The following morning, the primary antibody was aspirated, and inserts were rinsed with 500 μl of PBS for 5 min 3 times at room temperature. The PBS (pH = 7.4) was aspirated and then the inserts were probed with TRITC-labeled goat anti-rabbit antibody (1:500 in ADB; Jackson ImmunoResearch Laboratories Inc., West Grove, PA, USA) for 1 h at room temperature. After incubation with antibodies, preparations were mounted on slides using ProLong DAPI Antifade mounting medium containing 4′,6-diamidino-2-phenylindole (DAPI) for nuclear visualization (ThermoFisher Scientific, Carlsbad, CA, USA).

Immunohistochemistry and immunolocalization of Ca_V_1 in sectioned whole gill epithelia of rainbow trout were performed as follows. Whole gill arches were dissected out in PBS as described above (pH = 7.7), and fixed in Bouin’s solution for 4 h at room temperature. Following fixation, gill arches were rinsed and stored in 70 % ethanol at 4 °C until further processing. Fixed tissues were dehydrated through an ascending series of ethanol rinses (70–100 %), cleared with xylene and infiltrated and embedded in Polyfin Tissue Embedding Medium (cat. no. 50–279–84, Fisher Scientific). Then, 5-μm thick sections were cut using an HM 355 S rotary microtome (MICROM International GmbH, Walldorf, Germany), collected on glass slides and incubated overnight at 45 °C on a slide warmer. Sections were deparaffinized with xylene, rehydrated to water via a descending ethanol series (100 % to 50 %, then water), and subjected to heat-induced epitope retrieval in sodium citrate buffer (10 mM, pH = 6.00) heated in a microwave oven to 92–95 °C. The solution was allowed to cool for 20 min, reheated again, and cooled further for 15 min. Slides were then washed 3 times with PBS (pH = 7.4) and quenched for 30 min in 3 % H_2_O_2_ in PBS (pH = 7.4). Following quenching, slides were washed in 0.4 % Kodak Photo-Flo 200 in PBS (pH = 7.4) (PF/PBS, 10 min), 0.05 % Triton X-100 in PBS (pH = 7.4) (PBT, 10 min), and 10 % antibody dilution buffer (ADB: 10 % goat serum, 3 % BSA in PBT) in PBS (pH = 7.4) (ADB/PBS, 10 min), successively. Slides were incubated overnight at room temperature with rabbit polyclonal anti-Ca_V_1 (1:400 dilution in ADB). Following overnight incubation, sections were successively washed with PF/PBS, PBT, and ADB/PBS (10 min each) as described above, and incubated with tetramethylrhodamine isothiocyanate (TRITC)- labeled goat anti-rabbit antibody (1:500 in ADB; Jackson ImmunoResearch) for 2 h at room temperature. Slides were then washed in PF/PBS, PBT and PF/PBS (10 min each) and rinsed 3 times with 0.4 % PF in distilled water (PF/dH2O, 1 min each). Slides were air dried for 1 h and mounted with Molecular Probes ProLong Antifade (ThermoFisher Scientific) containing DAPI. Fluorescence images were captured using a Nikon Eclipse CI-S microscope and Nikon DS-Qi2 camera, and merged using ImageJ software (US National Institutes of Health, Bethesda, MD, USA).

### 2.8. Pharmacological inhibition of Ca_V_1

Since transcript abundance of many Ca_V_1 isoforms increased following FW exposure, a separate set of cultured gill epithelia was grown, and exposed to FW as described above. Half the inserts were treated with nifedipine (Sigma-Aldrich), an inhibitor of voltage-gated Ca_V_1 channel, along with apical FW exposure. Since immunolocalization results indicated the presence of Ca_V_1.3 in the apical membrane of epithelia, we added nifedipine to the apical side of cultured epithelia together with FW. Stock solution of nifedipine, was prepared in dimethyl sulfoxide (DMSO) and stored frozen until use, defrosted on the day of the experiment and diluted in FW to 0.1 μM (final DMSO concentration in apical media did not exceed 0.01 % *v*/v). TER was measured as described above directly prior to and immediately after the introduction of apical FW (or apical FW with nifedipine), as well as every 30 min for 2 h following.

### 2.9. Statistical analysis

**Fig. 1.**
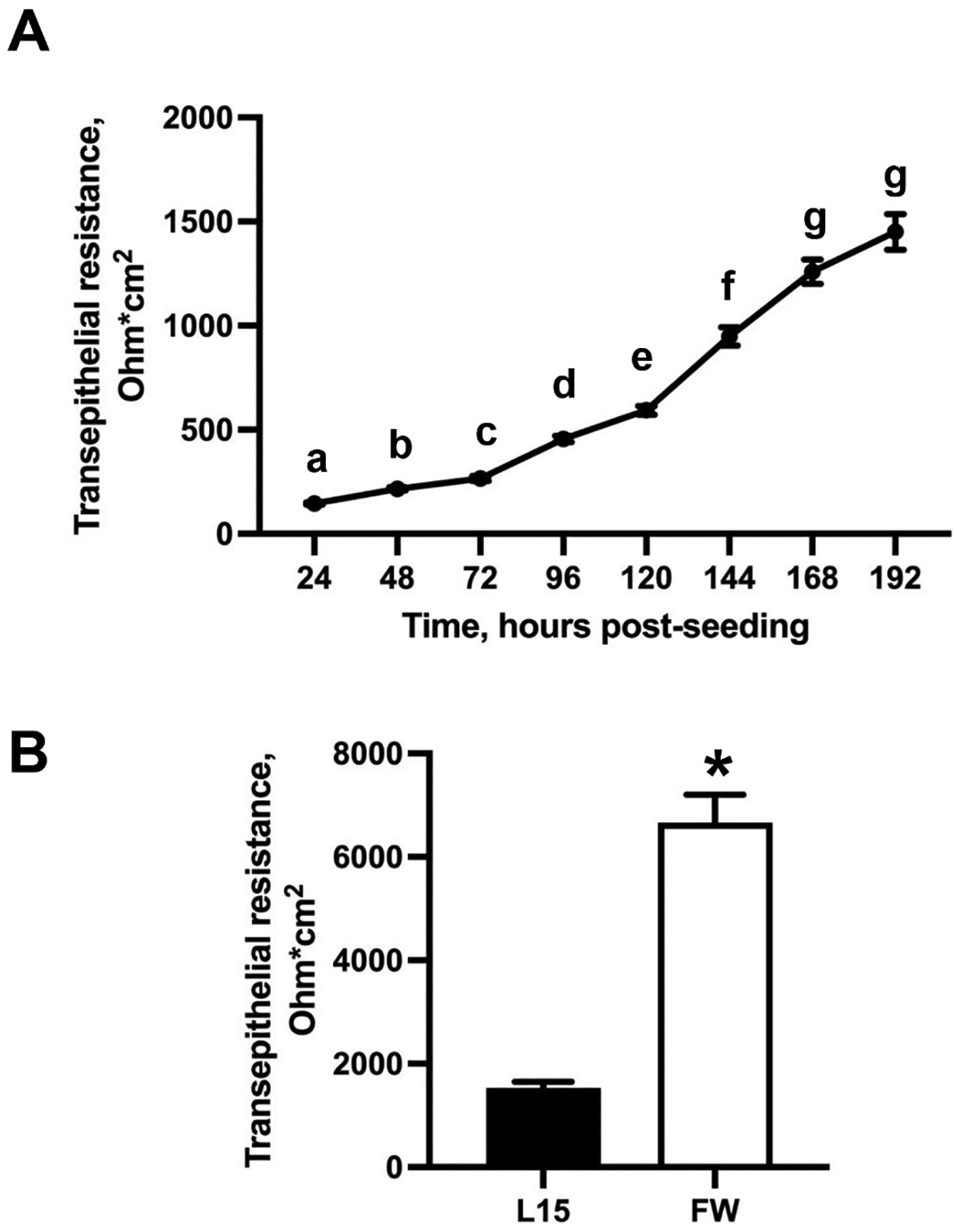
Transepithelial resistance (TER) of cultured trout gill epithelia in cell culture inserts. (A) The TER increases more rapidly after approximately 72 h (about 4 days) post-seeding and plateaus after approximately 168 h (about 1 week in culture) post-seeding. (B) The bar graph compares TER between L-15 media (L15) and freshwater (FW) exposed epithelia. TER has a significant difference after cells have been exposed to FW.

**Fig. 2.**
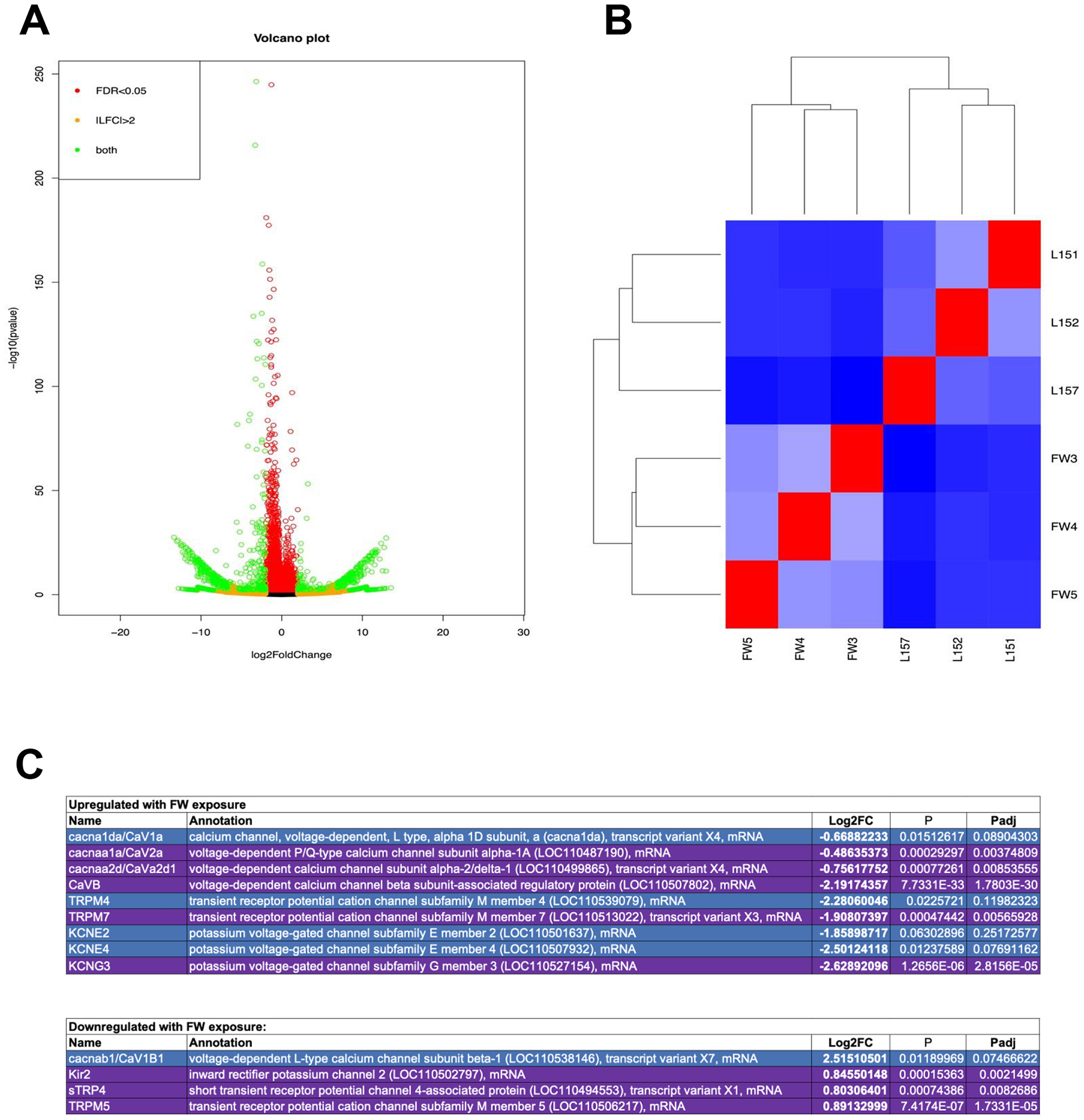
Summary of lab-generated RNAseq date for the current study. (A) A volcano plot of all transcripts expressed in cultured fish gill epithelia and changes in their mRNA abundance in response to freshwater exposure. The upregulated (right) and downregulated (left) transcripts with *p*-value (y-axis). The differential expression of the individual transcripts was determined by the fold-change (FC) in a DEseq2 analysis in Galaxy. (B) The heatmap demonstrating total gene expression differences between epithelia exposed to freshwater (FW) and cultured under symmetrical L-15 media conditions (L15). Blue-red continuum denotes similarity in gene expression. (C) Voltage-gated ion channels expressed in primary cultured gill epithelia of rainbow trout and changes in their transcript abundance following freshwater exposure. Note the presence of voltage-gated K^+^, transient receptor potential, and voltage-gated Ca^2+^ channels. The Log_2_FC and p-value were determinants of significant changes in up- (purple) and down-regulated (blue) abundance.

All statistical analyses were completed in either R v. 4.4.1 (RNAseq Deseq2 package release 1.44.0) or in GraphPad Prism version 10.4.1, as indicated below and following standard checks for normality and homogeneity of variance. Significant changes in TER during development of resistive properties (Fig. 1A, Fig. 6A) was assessed with a one-way ANOVA with a significance limit of *p* < 0.05. Significance of the effect of FW exposure on TER (Fig. 1B), and mRNA abundance of ion channels (Fig. 4) was assessed with Student’s *t*-test with a significance limit of p < 0.05. Significance of differential expression of transcripts in primary cultured epithelia exposed to FW (Fig. 2, Supplementary Table S1) was assessed using a Deseq2 package in R. The effects of pharmacological inhibition of Ca_V_1 on transepithelial resistance (Fig. 6B) were assessed using a two-way ANOVA isolating for the effects of time and FW exposure/treatment, and the interaction between the two factors, coupled with paired *t*-tests in GraphPad Prism, all with significance limit of p < 0.05.

## 3. Results

### 3.1. Culture of primary epithelia from rainbow trout

The transepithelial resistance (TER) of cultured gill epithelia increased significantly in 24-h increments until through 168 h (7 days) post-seeding (one-way ANOVA coupled with Tukey’s post-hoc test, *p* < 0.0001) (Fig. 1A). Apical exposure (water-facing side) of insert-cultured epithelia to FW resulted in a significant increase in TER from 1533 ± 115 Ω*cm^2^ to 6663 ± 539 Ω*cm^2^ within 2 h of exposure (two-tailed Student’s *t*-test, p < 0.0001) (Fig. 1B).

### 3.2. Transcriptome of cultured gill epithelia and VGIC transcripts

RNAseq analysis of cultured gill epithelia indicated that apical FW exposure resulted in a massive transcriptional response with upwards of 6600 transcripts changing in abundance within 2 h (Fig. 2A–B, Supplementary Table S1). A total of 50 VGIC transcripts were detected in the epithelium, including *Ca*_*V*_, *TRPM*, *Kir*, *KCNA*, *KCNC*, *KCND*, *KCNE*, *KCNG*, *KCNH*, *KCNQ*, *KCNS*, and *KCNV* channels (Supplementary Table S1). A total of 13 VGICs changed in transcript abundance in response to apical FW exposure (Fig. 2C).

Specifically, RNAseq indicated that pore-forming α-subunits of voltage-gated Ca^2+^ (Ca_V_) channels Ca_V_1 and Ca_V_2 anchoring Ca_V_ subunit *α2δ1*, as well as voltage-gated K^+^ channels KCNE2, KCNE4, KCNG3, and TRP channels TRPM4 and TRPM7 all increased in mRNA abundance with FW exposure, with mRNA abundance changes ranging from a 1.4-fold to 6.2-fold. In contrast, mRNA encoding Ca_V_1 subunit *β*, as well as inward-rectifier K^+^ channel Kir2, sTRP4 and TRPM5 decreased in abundance. It is worth noting that in addition to the abundance of VGICs (the focal point of this study), transcript abundance changes in ion transporters, bi-cellular and tricellular tight junctions, receptors, ligand-gated ion channels and gap junctions were observed (the reader is directed to Supplementary Table S1 for details).

### 3.3. VGICs are expressed in primary cultured gill epithelia of O. mykiss

**Fig. 3.**
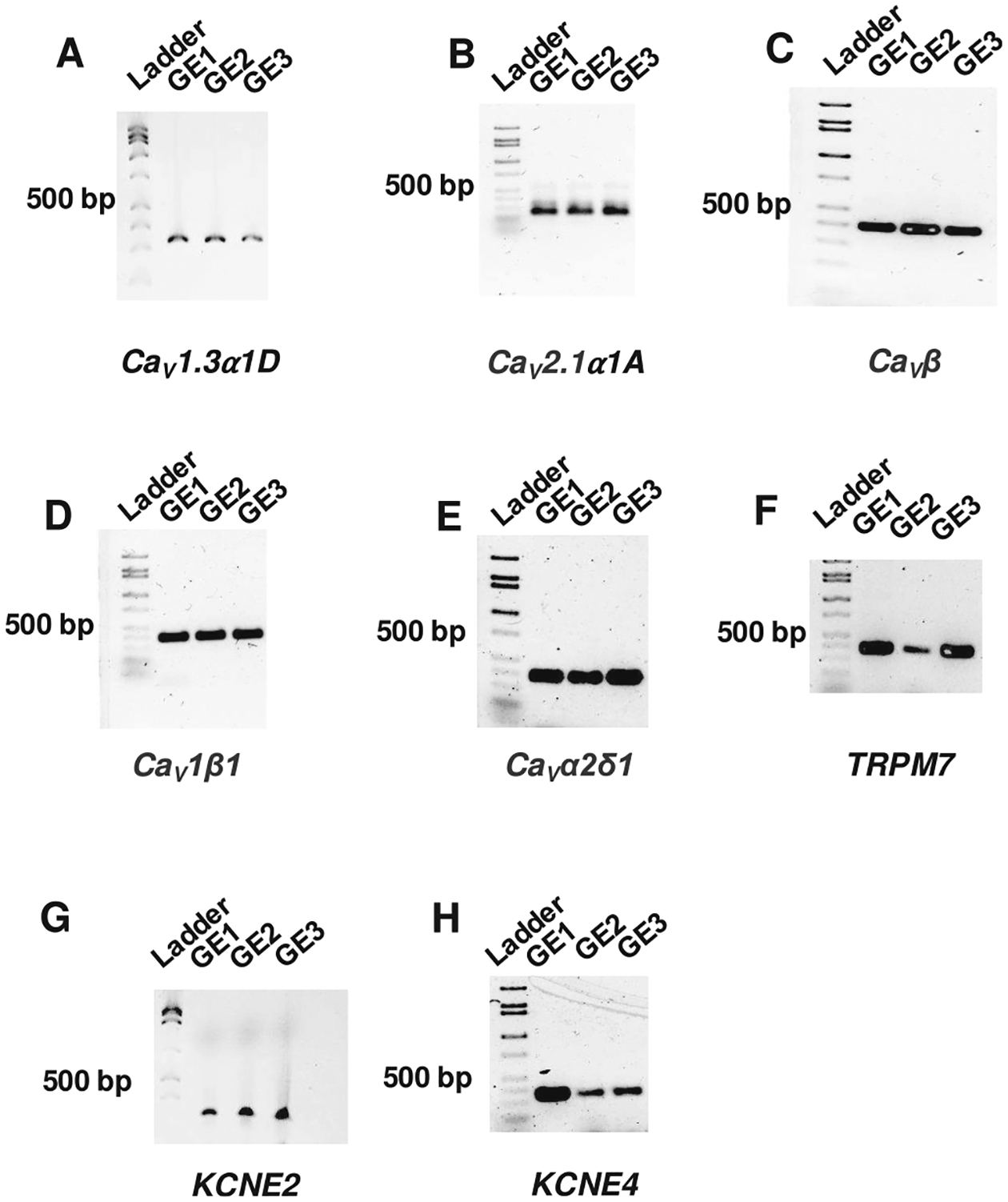
Direct confirmation of VGIC expression in cultured gill epithelia of *O. mykiss*. RT-PCR detection of transcripts encoding rainbow trout (A) *Ca V1.3α1D*, (B) *Ca*_*V*_*2.1α1A*, (C) *Ca*_*V*_*β*, (D) *Ca*_*V*_*1β*, (E) Ca_V_ *α2δ1*, (F) *TRPM7*, (G) *KCNE2*, (H) *KCNE4*. The 500 base-pair marker is denoted as a size reference in the ladder. Expression was confirmed in 3 biological replicates originating from different fish. GE – gill epithelia.

Since RNAseq data are prone to false positives, PCR and gel electrophoresis were used to confirm the expression of most VGICs using transcript-specific primers. Expression of *Ca*_*V*_*1.3α1D*, *Ca*_*V*_*2.1α1A*, *Ca*_*V*_*β*, *Ca*_*V*_*1β*, and Ca_V_ *α2δ1*, *TRPM7*, *KCNE2*, *KCNE4* was confirmed in three biological replicates of primary cultured trout gill epithelia (Fig. 3).

### 3.4. VGICs expressed in cultured gill epithelia altered in transcript abundance following apical FW exposure

**Fig. 4.**
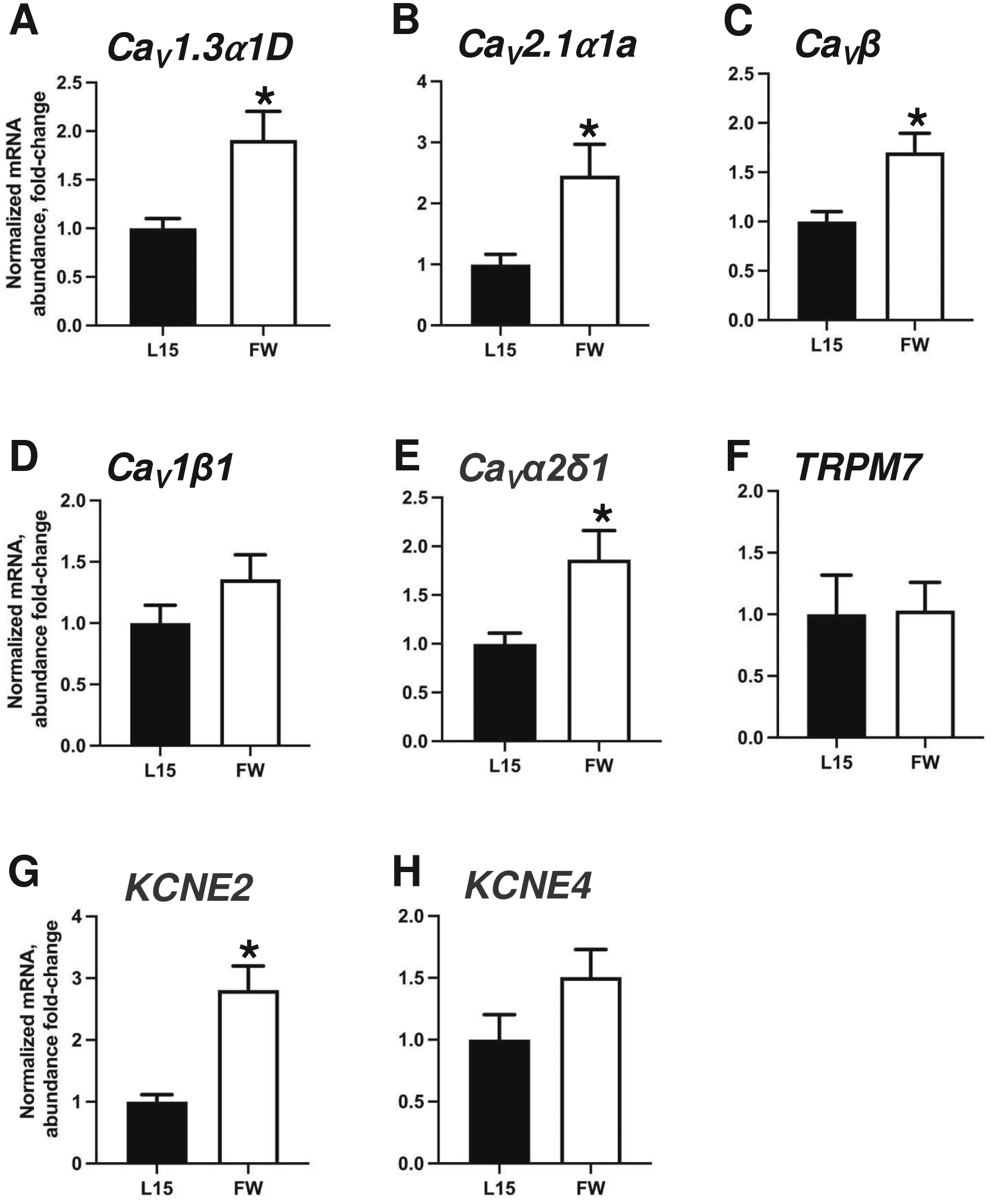
Transcript abundance of several VGICs altered in abundance following FW exposure. Transcript abundance of (A) α1D subunit of Ca_V_1.3, (B) α1A subunit Ca_V_2.1, (C) accessory Ca_V_ subunits β and (E) Ca_V_α2δ1, as well as (G) *KCNE2* increased in primary cultured gill epithelia exposed to FW. Transcript abundance of Ca_V_1 subunit β1, *TRPM7*, and *KCNE4* did not alter significantly. All data are presented at mean values ±SEM (*n* = 7–11). The asterisk indicates a significant difference due to FW exposure by Student’s *t*-test against L15 group (see text for *p*-values).

Quantitative real-time PCR was used to confirm transcript abundance changes observed in the RNAseq dataset. Following apical FW exposure for 2 h, transcript abundance of *Ca*_*V*_*1.3α1D* increased from 1.00 ± 10.12 to 1.92 ± 0.29 (Student’s *t*-test, *p* = 0.0249) (Fig. 4A), *Ca*_*V*_*2.1α1A* - from 1.00 ± 0.17 to 2.46 ± 0.51 (Student’s t-test, *p* = 0.0427) (Fig. 4B), *Ca*_*V*_*β* - from 1.00 ± 0.10 to 1.70 ± 0.19 (Student’s t-test, *p* = 0.0127) (Fig. 4C), and Ca_V_ *α2δ1* – from 1.00 ± 0.11 to 2.26 ± 0.48 (Student’s t-test, *p* = 0.0355) (Fig. 4E), *KCNE2* - from 1.00 ± 0.11 to 4.59 ± 0.98 (Student’s t-test, *p* = 0.0010) (Fig. 4G). Transcript abundance of *Ca*_*V*_*1β1* (Fig. 4D), *TRPM7* (Fig. 4F), and *KCNE4* (Fig. 4H) did not alter significantly following apical FW exposure.

### 3.5. Immunolocalization of Ca_V_1.3

**Fig. 5.**
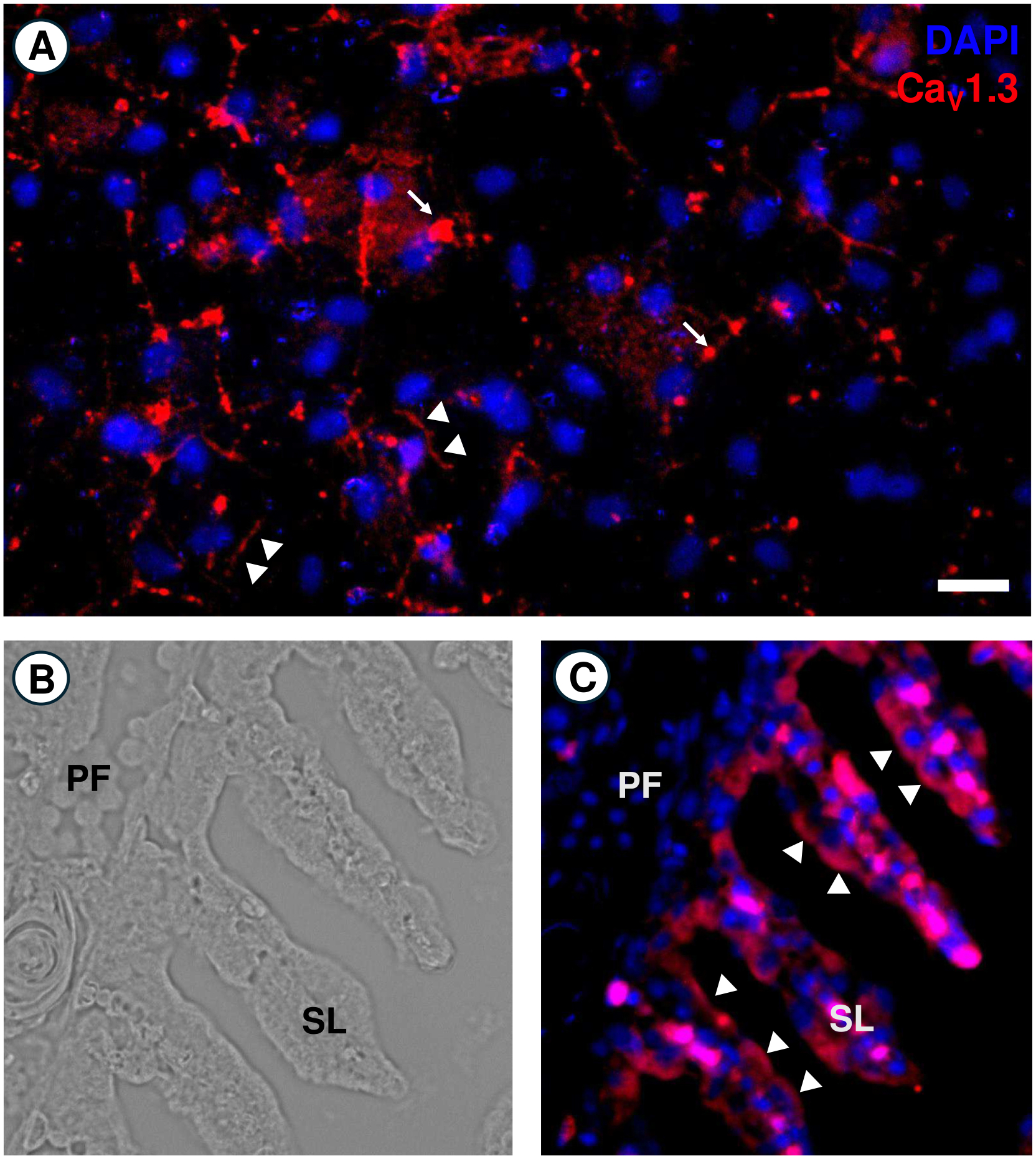
Immunolocalization of Ca_V_1.3 in cultured and intact gill epithelia of *O. mykiss*. (A) In cultured gill epithelia, punctate Ca_V_1.3 immunoreactivity (denoted by triangles) was observed across epithelial cells. Often, immunolocalization clustered in the cell-cell regions (denoted by arrows). (B–C) Bright-field and fluorescent images of longitudinally sectioned primary filaments of intact *O. mykiss* gill. Ca_V_1.3 was present on the apical water-facing side of secondary lamellae as well as primary filaments. PF - primary filament, SL - secondary lamellae. DAPI staining was used to visualize cell nuclei in blue. Scale bars Scale bars (A) = 50 μm. (B and C) = 10 μm.

We consistently noted transcript abundance changes of VGICs related to subunit assembly of voltage-gated ion channel Ca_V_1.3. Therefore, we undertook immunohistochemical analysis using a custom-made antibody and demonstrated the presence of Ca_V_1.3 in cultured gill epithelial cells with some immunoreactivity clustered around cell-cell contacts (Fig. 5A). In an intact gill filament, immunolocalization was observed in the apical side of both primary and secondary lamellae epithelia (Fig. 5B–C). Within primary lamellae, Ca_V_1.3 was present in the interlamellar spaces. Strong immunoreactivity was observed lining apical side of secondary lamellae epithelia (Fig. 5C).

### 3.6. Pharmacological inhibition of Ca_V_1 in FW-exposed epithelia increased TER

**Fig. 6.**
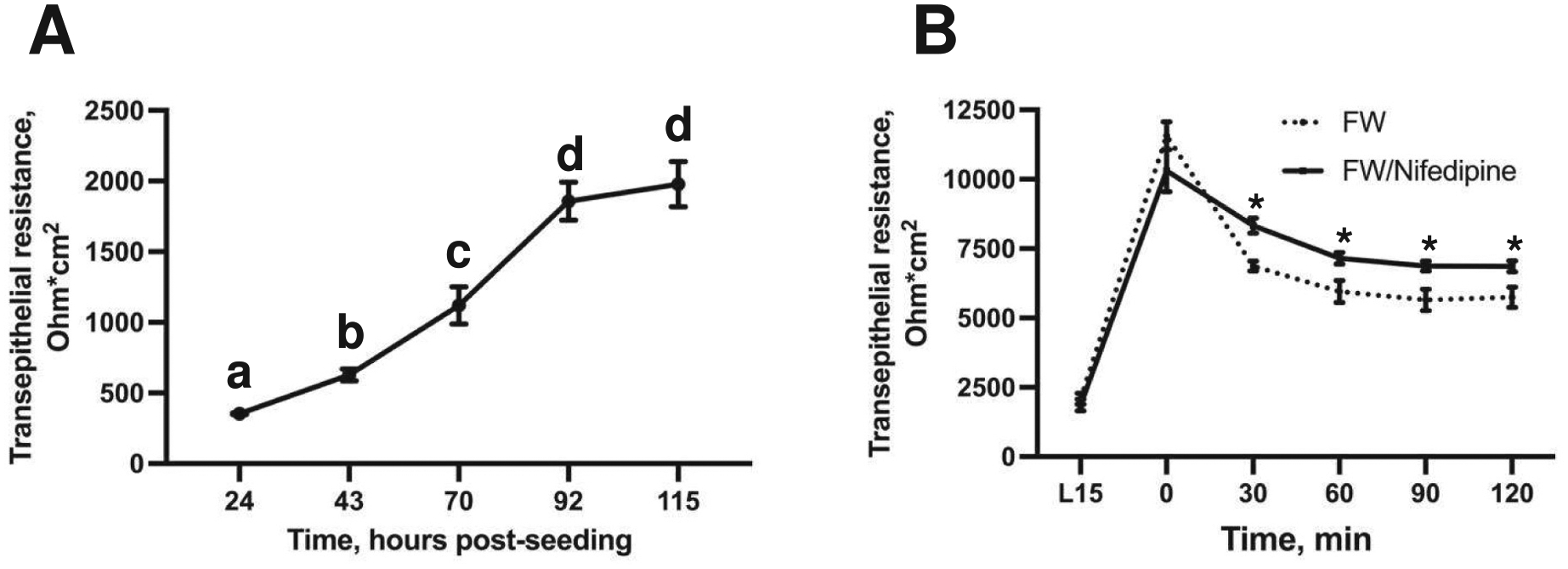
Effects of pharmacological inhibition of Ca_V_1 on response to apical FW exposure. (A) Cultured epithelia were once again grown until they developed stable resistive properties. (B) Epithelia were then exposed to FW, where pharmacological inhibition of Ca_V_1 resulted in increased TER compared with FW-cultured epithelia where Ca_V_1 remained functional. All values are reported as mean ± SEM (*n* = 9 in A, *n* = 5 in B). Significant differences determined by a one-way ANOVA coupled with post-hoc tests in A, and a two-way ANOVA coupled with *t*-tests in B.

mRNA abundance of Ca_V_1 subunits appeared upregulated in FW-exposed epithelia, and Ca_V_1.3 appeared immunolocalized to the apical side of gill epithelia. Therefore, we inhibited Ca_V_1 with nifedipine in cultured trout gill epithelia following apical exposure to FW. Cultured epithelia were once again grown until they developed stable resistive properties, significantly increasing in TER (one-way ANOVA *p* < 0.0001, coupled with Tukey post-hoc tests) (Fig. 6A) and exposed to FW, where pharmacological inhibition of Ca_V_1 resulted in increased TER compared to the FW-exposed epithelia where Ca_V_1 remained functional (two-way ANOVA p_time_ < 0.0001, p_treatment_ = 0.0075, p_interaction_ = 0.0020; coupled with *t*-tests) (Fig. 6B).

## 4. Discussion

### 4.1. Overview of findings

VGICs are expressed in epithelia of all animals examined to date, and primary cultured gill epithelia offer an excellent and convenient model for studying VGICs without layers of excitable tissues present in intact gills. In the current study, we provide a great resource of transcriptional changes in primary reconstructed fish gill epithelial model following FW exposure. We detected expression of many voltage-gated Ca^2+^, K^+^, Na^+^, and cation-selective channels. Several Ca^2+^ and K^+^-permeable VGICs were shown to have changed in transcript abundance in cultured gill epithelia following exposure to FW. Altered transcript abundance of voltage-gated Ca_V_1 channel subunits were of particular interest since Ca_V_1 has been connected to ion transport regulation in the Malpighian tubule epithelia of insects (MacPherson et al., 2001; Kolosov et al., 2021; Farrell et al., 2024). In at least one group of insects (lepidopteran larvae) active Ca_V_1 was shown to be necessary for membrane Ca^2+^ permeability, maintaining high intracellular Ca^2+^ levels, which in this epithelium led to increased K^+^ secretion (Kolosov et al., 2021). Using a custom-made anti-Ca_V_1.3 antibody, we demonstrated Ca_V_1.3 expression in both cultured and intact rainbow trout gill epithelia. Since many Ca_V_1 subunits were upregulated in response to FW exposure, we inhibited Ca_V_1 in cultured gill epithelia exposed to FW, which resulted in TER levels stably elevated above those of epithelia with intact Ca_V_1. Taken together, we demonstrate that VGICs are present in a primary cultured fish gill epithelium model and suggest that they may contribute to the regulation of epithelial ion transport and resistive properties in the face of FW exposure. We speculate that Ca_V_ channels may connect barrier and transport properties of the gill with autonomous osmosensing via Ca^2+^ signaling – a claim that will require further studies.

### 4.2. General autonomous transcriptomic response of cultured gill epithelia to FW

Apical FW exposure of cultured trout gill epithelia caused an increase in TER along with a massive transcriptional response, where many ion transporters (including VGICs, as well as ligand-gated and mechanosensitive ion channels) altered in mRNA abundance. This was accompanied by changes in mRNA abundance of tight junctions, gap junctions, and receptors. Of particular interest is upregulation of Na^+^/K^+^-ATPase and V-type H^+^-ATPase subunits, KCNK channel, and transcripts encoding scaffolding ZO-1, ZO-3, bicellular tight junction proteins claudins 1, 4, and 7b, and tricellular tight junction proteins Tric, LSR and ILDR1 (Supplementary Table S1). In contrast, carbonic anhydrase 4, H^+^/Cl^−^ exchange transporters 3 and 5, Na^+^/H^+^ exchanger 6, several V-type H^+^-ATPase subunits all decreased in transcript abundance. Transcellular and paracellular components of gill ion transport and barrier function have been firmly linked with response to external salinity changes in the intact and cultured trout gill epithelia (Chasiotis et al., 2012; Gilmour, 2012; Kolosov and Kelly, 2013; Kolosov and Kelly, 2016; Kolosov and Kelly, 2018; Kovac and Goss, 2024). These transcriptional changes are indicative of increased active ion uptake and decreased paracellular permeability, coupled with an attempt to decrease cell volume in the face of FW exposure - in agreement with a sharp increase in TER in FW-exposed epithelia (Fig. 1B).

Ca^2+^-activated Cl^−^ channels Tweety and anoctamin 9, as well as Ca^2+^-activated K^+^ channels all decreased in mRNA abundance, and volume-activated anion channel increased in mRNA abundance. Changes in transcript abundance of volume- and Ca^2+^-activated K^+^ and Cl^−^ transporters may be a part of a regulatory cell volume decrease response or may be aimed at direct ion uptake in FW-exposed epithelia.

Of additional interest to the reader may be downregulation of β3a adrenergic receptor and atrial natriuretic peptide receptor in the cultured epithelia exposed to FW, as well as increased abundance of thyroid hormone receptor β. Thyroid hormone and natriuretic peptides have been implicated in the regulation of active ion transport and paracellular permeability in the fish gill (Loretz and Pollina, 2000; Deal and Volkoff, 2020; Kolosov and Kelly, 2020). β3a adrenergic receptors are a part of salinity change stress response linked to active Na^+^ uptake in FW, perhaps anticipating action of stress hormones on peripheral tissues following salinity acclimation (Kumai et al., 2012). These receptor abundance changes were induced in absence of, but perhaps in anticipation of, systemic endocrine input set to address altered cellular salt and water balance.

### 4.3. What is the function of VGICs in the fish gill epithelium?

Animal epithelia benefit from molecular mechanisms that autonomously detect and respond to environmental salinity changes, prior to systemic endocrine responses. The use of VGICs could, in principle, allow animal epithelia to rapidly detect environmental (or systemic) changes in ion and water content. VGICs are: (i) permeable to ions, (ii) can be opened/closed by changes in membrane potential (which in epithelia correspond tightly with directional ion transport), and (iii) can be activated by upstream agents that change membrane potential. The roles of VGICs in excitatory tissues (muscles and nerves) have been investigated in detail in many animal groups (e.g., Catterall, 2011; Wang et al., 2017; Senatore and Spafford, 2022), but how VGICs regulate ion transport and barrier properties in epithelia of fishes has not been characterized to date.

In the current study, we isolated and cultured gill epithelial cells from rainbow trout to circumvent structural heterogeneity and architectural complexity of the intact fish gill, and to enable examination of VGICs present specifically in the gill epithelium. Based on previous research, VGICs have been found in the epithelia of every living animal examined ranging from bivalves to humans (reviewed in Kapoor et al., 2021; Dates and Kolosov, 2024). We successfully detected the expression of several groups of VGICs in the cultured gill epithelia of rainbow trout. Like the previous studies on insect epithelia (Kolosov et al., 2019; Kolosov and O’Donnell, 2019; Kolosov et al., 2021), cultured fish gill epithelia express HCN channels, a multitude of voltage-gated K^+^ channels (K_V_), as well as full subunit assemblies of Ca_V_ and Na_V_ channels (see Supplementary Table S1, *VGICs*).

One of the limitations of the current study for enumeration of all VGICs expressed in trout gill epithelia is that this model is a pavement cell-only culture, with no mitochondrion-rich cells present. Thus, it is likely that more VGICs than are disclosed in the current study exist in the trout gill epithelium. Previous studies have shown marked differences in transcript abundance and resistive properties between pavement and mitochondrial-rich cells in fish gills (Chasiotis et al., 2012; Kolosov et al., 2014; Lai et al., 2015; Schnell et al., 2016).

Only a subset of all VGICs detected in cultured trout gill epithelia showed altered mRNA abundance in gill epithelia following exposure to FW belonged to KCNE, TRPM, and Ca_V_ families of ion channels. KCNE proteins form heteromeric complexes with pore-forming K_V_ α subunits modulating their function. In the current study, KCNE2 increased in mRNA abundance. In previous studies, KCNE2 has been detected in choroid plexus and gastric and thyroid epithelia of mice, where it plays a role in the transport of K^+^ and other solutes (Abbott, 2015). Specifically, KCNE2 has been shown to alter functional attributes of KCNH2 (Abbott et al., 1999), KCNQ1 (Abbott, 2014). In the current study, we also detected KCNA, KCNC, KCND, KCNG, KCNH, KCNQ, KCNS, and KCNV types of K_V_ channels in the cultured trout gill epithelia. For instance, in mouse stomach epithelia, KCNE2/KCNQ1 pairing acts as an apical recycling K^+^ conduit activated by low external pH, enabling gastric acid secretion in mammalian kidney COS cells (Heitzmann et al., 2007). In choroid plexus epithelia of mice, apical KCNE2/KCNQ1 pairing was implicated in controlling K^+^ content of the cerebrospinal fluid (Roepke et al., 2011). KCNQ1 has been found in Malpighian tubule epithelia of caterpillars and larval mosquitoes, and was implicated in epithelia K^+^ transport as well (Kolosov et al., 2019; Kolosov and O’Donnell, 2019; Farrell et al., 2024). Thus, in principle, increased abundance of KCNE2 in FW-exposed gill epithelia could modulate the function of the multitude of endogenous K_V_ channels present in this epithelium.

In general, TRP channels are voltage-dependent channels that are permeable to cations. TRP channels have been found in epithelia previously (Farrell et al., 2024; Dates et al., 2024) and are known to play a key role in Ca^2+^ signaling and directing signaling pathways (Fleig and Penner, 2004). TRPM5 is a Ca^2+^-activated non-selective monovalent cation channel (Chubanov et al., 2024). While no change was observed in the mRNA abundance of TRPM7 channels, they are permeable to Ca^2+^ and may contribute to Ca^2+^ homeostasis and signaling in the fish gill epithelium (Jin et al., 2011; Fleig and Chubanov, 2014). For instance, zebrafish with mutations of TRPM7 develop kidney stones, highlighting a possible role of this channel in epithelial function (Elizondo et al., 2005).

Ca_V_ channels are expressed in non-excitable tissues of animals where they play a variety of roles, from directional Ca^2+^ transport to intracellular signaling (Pitt et al., 2021). In epithelia, Ca_V_1 channel has been shown to regulate ion transport in the Malpighian tubules of insects (Kolosov et al., 2021; Farrell et al., 2024). In the present study, pore-forming α subunits of Ca_V_1.3 and Ca_V_2.1 were both upregulated with exposure to FW. Ca_V_1.3 in the fish gill is located on the apical side of the gill epithelium and may provide an additional avenue for the regulation of intracellular Ca^2+^ levels by allowing for entry point of environmental Ca^2+^ into the cell, and may participate in the regulation of directional ion transport and the regulatory cell volume decrease previously reported in FW-exposed cultured trout gill epithelia (Leguen and Prunet, 2001; Leguen and Prunet, 2004). The latter in epithelia has been proposed to be preceded by an increase in cytosolic Ca^2+^ levels via influx from Ca^2+^-permeable TRP channels (Larsen and Hoffmann, 2020). Auxiliary subunits of Ca_V_1 - β and α2δ - modulate channel kinetics, gating and membrane trafficking (Borowik and Colecraft, 2022; Dolphin and Obermair, 2022). Changes in subunit Ca_V_ channel mRNA abundances may indicate that as the epithelium restructures and responds to apical FW exposure, potentially engaging Ca_V_ channels in regulatory volume decrease. Kinetics and gating of Ca_V_ channels may also change upon exposure to apical FW.

### 4.4. Does Ca_V_1.3 in the fish gill epithelium face water (apical) or blood (basolateral)?

In addition to changing mRNA abundance with FW exposure, we found that Ca_V_1.3 was present in most epithelial cells. However, immunoreactivity often clustered beside cell-to-cell barriers. In addition, in this study cross sections of the gill were used to ascertain the localization of Ca_v_1.3 was shown along the apical membrane, i.e., facing water on the outside.

### 4.5. What is the function of Ca_V_1.3 in the fish gill epithelium?

Exposure of epithelia to FW resulted in an initial increase in TER, followed by a slightly lower plateau, which is in agreement with previous studies on cultured fish gill epithelia (e.g., Wood and Pärt, 1997; Wood et al., 1998; Kelly and Wood, 2002). FW used in the current study was quite ion-poor, which undoubtedly influenced the electrophysiological properties of the cultured epithelia as previous studies reported that ion content of FW has an effect on the resistive properties of the culture epithelia, and potentially Ca_V_1 dynamics in FW-exposed cultured epithelia (Kelly and Wood, 2008; Chasiotis et al., 2012). When Ca_V_1 was inhibited in epithelia exposed to FW, TER ended up plateauing significantly higher. This indicates that Ca_V_1 channels may be crucial for the restructuring of paracellular junction permeability and/or active ion transport in cultured trout gill epithelia exposed to apical FW.

Ca_V_1.3 channels are known to activate at inside-negative membrane voltages (Helton et al., 2005). Therefore, Ca_V_1.3, upregulated with FW in the current study, specifically, can react to changes in membrane potential and changes in salinity surrounding that of FW, to provide a direct link to regulation of resistive properties (which includes tight junction restructuring and potential changes in ion transport) of the cultured gill epithelia. Furthermore, Ca_V_1.3 has been shown to affect ion transport and tight junction permeability in several model epithelia. Ca_V_1.3 and a rise in [Ca^2+^]_i_ play a role in the mechanism of osmotic stress-induced restructuring of intestinal epithelial tight junction barrier (Samak et al., 2011). Ca_V_1 has been shown to regulate ion transport in the Malpighian tubules of larval insects (Kolosov et al., 2021; Farrell et al., 2024). Therefore, upregulating Ca_V_1.3 in the gill epithelium may be the cell’s way of attuning ion transport and junctional permeability with changes in environmental salinity and in response to osmotic stress.

Ca_V_1.3 is less abundant (in the current study) in L-15 cultured epithelia because its functional maximum would not be useful in more saline waters. Increasing environmental salinity and increasing ion levels would necessitate that other Ca_V_ channels be utilized that require a more significant membrane depolarization to open, which would be easier to achieve in the light of a smaller ion concentration gradient. The amount of Ca^2+^ coming into the cell would depend on the Ca^2+^ concentration gradient between the inside of the cell and the water, the apical membrane potential, and the number of channels open. Fluctuating Ca^2+^ levels, in turn, can offset directional ion transport and tight junction permeability. Primary cultured epithelia of rainbow trout have been reported to exhibit a balance influx/efflux of Ca^2+^ under symmetrical L15/L15 conditions, where direction and magnitude of Ca^2+^ changed depending on the salt content of the apical media (Kelly and Wood, 2008).

**Fig. 7.**
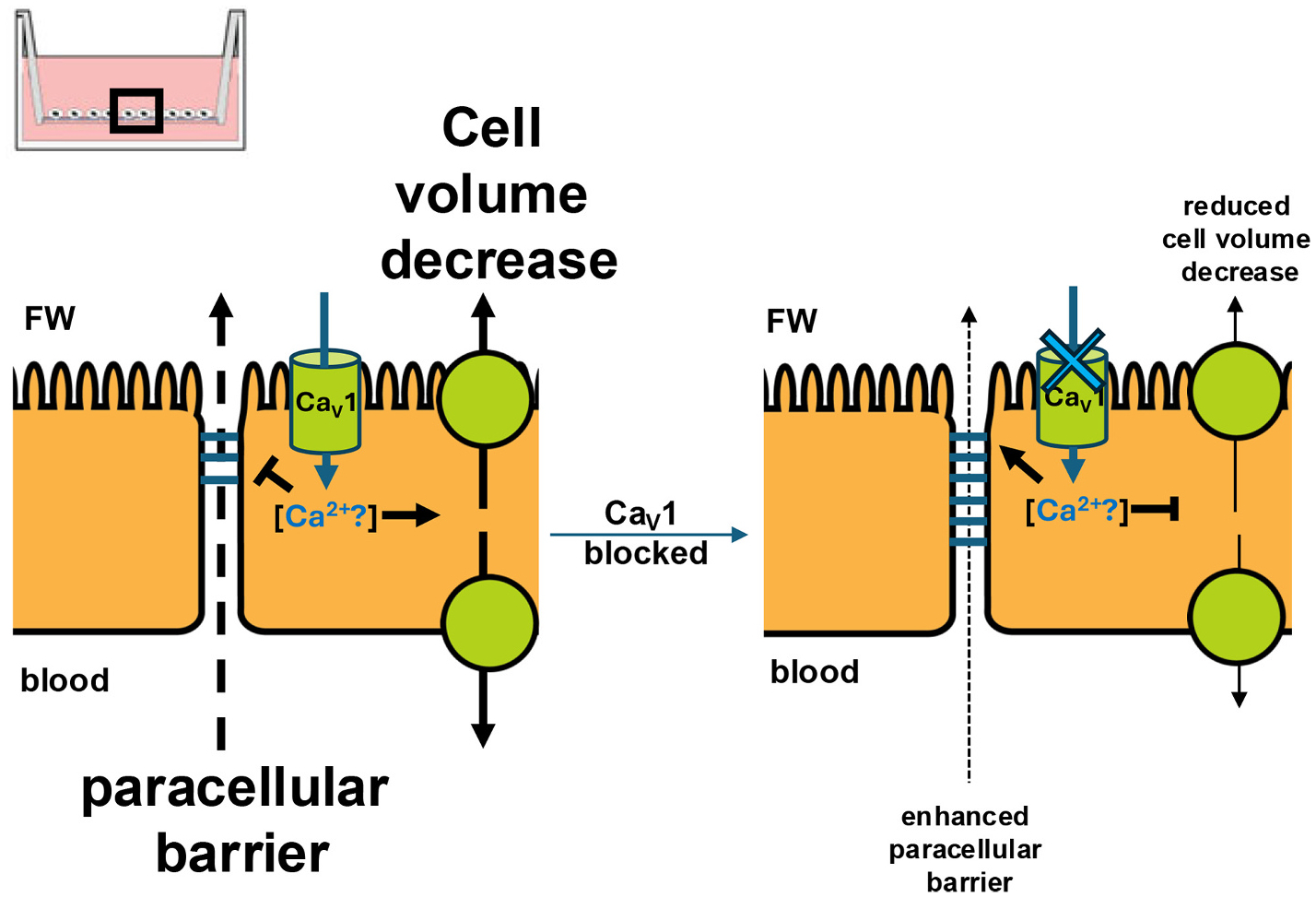
Ca_V_1.3 regulates barrier, ion transport, and volume regulation in the fish gill epithelium. Ca_V_1.3 in the gill pavement cells may provide a connection between external salinity, membrane potential, Ca^2+^ flux into the cell, and [Ca^2+^]_i_ levels, which in turn can influence tight junction permeability, directional ion transport, and regulatory cell volume changes. Dashed black lines indicate passive leakage of ions. Solid black lines indicate active ion transport. Solid blue indicates Ca^2+^ movement.

Together, our data show that Ca_V_1.3 in the gill pavement cells of cultured trout gill epithelia may provide a connection between external salinity, membrane potential, Ca^2+^ flux into the cell, and [Ca^2+^]_i_ levels (Fig. 7), which in turn may be indicative that Ca_V_1 may be able to influence tight junction permeability, directional ion transport, and regulatory cell volume changes. The claim that Ca_V_1 is involved in regulatory volume changes in strengthened by the fact that multiple Ca^2+^-gated cation and anion channels (e.g., Piezo, VRAC, Tweety, anoctamins) typically involved in regulatory volume changes altered in transcript abundance with FW exposure in the current study as well (see Supplementary Table S1).

### 4.6. Future directions and conclusions

Future studies will focus on loss-of-function and gain-of-function methodology to further delineate the role of Ca_V_ channels in the fish gill epithelium, differences in expression and abundance of VGICs between fish gill pavement cells and mitochondrion-rich cells. *What does Ca*_*V*_*2 do in the fish gill epithelium? Do Ca*_*V*_ *channels expressed epithelia remain voltage-gated?* Alternative splicing and exon use have been shown to alter voltage-dependence of Ca_V_ channels (Huang et al., 2022). *Does activation/inactivation of Ca*_*V*_ *channels alter intracellular Ca*^*2*+^ *levels?* To firmly establish the connection between Ca_V_ channels and intracellular Ca^2+^ levels in the fish gill epithelium similar to that observed in epithelia of other animals, intracellular Ca^2+^ levels will need to be monitored along with loss-of-function and salinity exposure experiments.

## Supplementary Material

1

## Figures and Tables

**Table 1:** Primer sets used for RT-PCR and qPCR analysis of voltage-gated ion channel transcript expression and abundance in primary cultured gill epithelia of rainbow trout *Oncorhynchus mykiss*. T_a_ – annealing temperature.

Name	Forward Primer	Reverse Primer	NCBI Accession Number	NCBI Accession Number	Ta
Elongation Factor ala	GGCAAGTCAACCACCACAG	GATACCACGCTCCCTCTCAG		AF498320.1	60
Cav1.3α1D	CTCATCGCCTTCAAACCCAG	CACGCCAACATGATCTCCTG		XM_036948175.1	63
Cav2.1α1a	ACATCGACACACAACACTGC	ACACTAGCCTCACACACCAA		XM_036948345.1	57
Cavβ	CACTGGTCCTGCTGTCTGTC	CGCATTCTTCAGGTGGTGGA		XM_021588109.2	59
Cavlβl	GACACAGAGCAACACACACA	GGGAAGTAAACTGGACACCG		XM_036938211.1	57
Cavα2δ1	GGGCCAGTAACCGAAATGTC	AATCCACACAGAGAGCACCA		XM_036957132.1	60
TRPM7	CCTCCTTCCTCCATCCAGAC	GGTGAAGGGGATGCTCTGAG		XM_036964068.1	61
KCNE2	CAGTGGTGGAAAAGTACAGA	ATCTGGCAGACTCACTAAAC		XM_021579336.2	54
KCNE4	CTTTTCATTCCACCTCAAACTCC	CCTTCCTTCATTGACATCCTGG		XM_021588354.2	61

## Data Availability

Data will be made available on request.

## Appendix A. Supplementary data

Supplementary data to this article can be found online at https://doi.org/10.1016/j.cbpa.2025.111835.
